# EHMTI-0135. Migraine outcomes and cortical excitability: analysis of pattern-reversal visual evoked potentials (PR-VEP)

**DOI:** 10.1186/1129-2377-15-S1-E34

**Published:** 2014-09-18

**Authors:** A Sergeev, E Snopkova, G Tabeeva, V Osipova

**Affiliations:** 1 Department of Neurology, I.M. Sechenov First Moscow State Medical University, Moscow, Russia

## Introduction

Outcomes of migraine (M) in the elderly as well as age-related neurophysiological changes are poorly studied.

Aim of study was to evaluate cortical excitability changes in M patients depending on patient’s age and M outcome.

## Methods

Study groups comprised: 20 young (aged 20-49, YM) and 35 elderly pts (aged 50-70, EM) with ongoing typical M attacks; 11 elderly pts (aged 50-70) with complete M cessation by now and typical M course in the past (No more M) and 7 pts of any age with partial M cessation (preservation of M aura without headache) - Late Life Migraine Equivalents (LLME). Diagnosis was based on ICHD-2, 2004. Control groups: healthy subjects without headache (n=15 aged 20-49, n=15 aged 50-70). The RP-VEP test was carried out in a period free from migraine attacks.

## Results

Significant increase in total N75-P100 (p<0,05) and more marked dyshabituation were obtained in YM (7,74±2,7 and -8.1%), EM (7,85±3,9 and -2.3%) and LLME groups (8.7±2.5 and -0.76%) compared to group no more M (5.6±1,5) and both control groups without M anamnesis (6,85±2,6 and -14.4%; 6,24±2,6 and -15.1%).

## Conclusions

Our study has shown that patients with active M and those keeping M aura but shedding headache phase (LLME) demonstrate neurophysiological pattern typical for M reflecting cortical hyperexcitability which could be the basis for M preservation in any age. On the contrary, in patients of any age with complete M cessation we revealed complete normalization of both indexes which appeared comparable to subjects who never suffered from M.

No conflict of interest.

